# Predicting the Effects of Coastal Hypoxia on Vital Rates of the Planktonic Copepod *Acartia tonsa* Dana

**DOI:** 10.1371/journal.pone.0063987

**Published:** 2013-05-17

**Authors:** David T. Elliott, James J. Pierson, Michael R. Roman

**Affiliations:** University of Maryland Center for Environmental Science (UMCES), Horn Point Laboratory, Cambridge, Maryland, United States of America; University of Connecticut, United States of America

## Abstract

We describe a model predicting the effects of low environmental oxygen on vital rates (egg production, somatic growth, and mortality) of the coastal planktonic copepod *Acartia tonsa*. Hypoxic conditions can result in respiration rate being directly limited by oxygen availability. We hypothesized that *A. tonsa* egg production, somatic growth, and ingestion rates would all respond in a similar manner to low oxygen conditions, as a result of oxygen dependent changes in respiration rate. Rate data for *A. tonsa* egg production, somatic growth, and ingestion under low environmental oxygen were compiled from the literature and from supplementary experiments. The response of these rates to oxygen was compared by converting all to the analogous units in terms of oxygen utilization, which we termed analogous respiration rate. These analogous respiration rates, along with published measurements of respiration rates, were used to parameterize and evaluate the relationship between *A. tonsa* respiration rate and environmental oxygen. At 18°C, our results suggest that *A. tonsa* experiences sub-lethal effects of hypoxia below an oxygen partial pressure of 8.1 kPa (∼3.1 mg L^−1^ = 2.3 mL L^−1^). The results of this study can be used to predict the effects of hypoxia on *A. tonsa* growth and mortality as related to environmental temperature and oxygen partial pressure. Such predictions will be useful as a way to incorporate the effects of coastal hypoxia into population, community, or ecosystem level models that include *A. tonsa*. This approach can also be used to characterize the effects of hypoxia on other aquatic organisms.

## Introduction

The occurrence and extent of coastal hypoxia has increased over the last several decades [1]. Hypoxia is often defined as dissolved oxygen concentrations of <2 mg L^−1^ (at 18°C in seawater = 1.5 mL L^−1^ = 5.6 kPa oxygen partial pressure), although more biologically relevant definitions may be needed [2], [3]. When exposed to hypoxia, aquatic organisms compensate for the reduced oxygen availability in several ways, including initial attempts to maintain oxygen delivery, followed by conservation of energy, and finally a reliance on anaerobic respiration during prolonged exposure [2], [4]. Physiological consequences of low oxygen include reduced feeding, reproductive success, and growth [2], [4]. At the extreme, exposure to hypoxia results in either mortality or emigration from the affected region [1], [3], [4].

Unlike benthic and demersal organisms, plankton are not restricted to living at or near the seabed, and may avoid hypoxic bottom water to varying extents. Although zooplankton may avoid hypoxic bottom water by migrating vertically upward in the water column, laboratory and field studies have demonstrated that some zooplankton do reside in hypoxic waters, in the open ocean, coastal regions, and freshwater [5], [6], [7]. Many zooplankton that reside in open ocean regions with persistent oxygen minimum zones have evolved specific mechanisms to survive daily excursions down into low-oxygen environments [7]. These animals display several strategies for tolerating exposure to extremely low oxygen conditions, including enhanced adenosine triphosphate (ATP) production prior to and following exposure to hypoxia, increased anaerobic ATP production, and decreased energy consumption through metabolic suppression [7], [8]. In contrast to oxygen minimum zones, coastal hypoxia is often seasonal and is a relatively recent phenomenon in many regions [1]. As a result, coastal zooplankton are less likely to have evolved such specialized mechanisms to tolerate exposure to low oxygen, and behavioral, rather than physiological adaptations appear to occur in response to hypoxia [9]. These coastal species may instead attempt to avoid hypoxic waters, and experimental evidence suggests that behavioral avoidance may be an adaptive trait in the common coastal copepod *Acartia tonsa*, occurring in some populations but not in others [10]. Overall, coastal zooplankton appear to be more likely to reside in hypoxic water when hypoxia occupies a large portion of the water column [11]. In any case, it is clear that zooplankton do occur in low oxygen coastal bottom waters [11], [12], [13], and that these animals may experience sub-lethal consequences or even mortality upon exposure to hypoxia [14], [15], [16].

**Figure 1:**
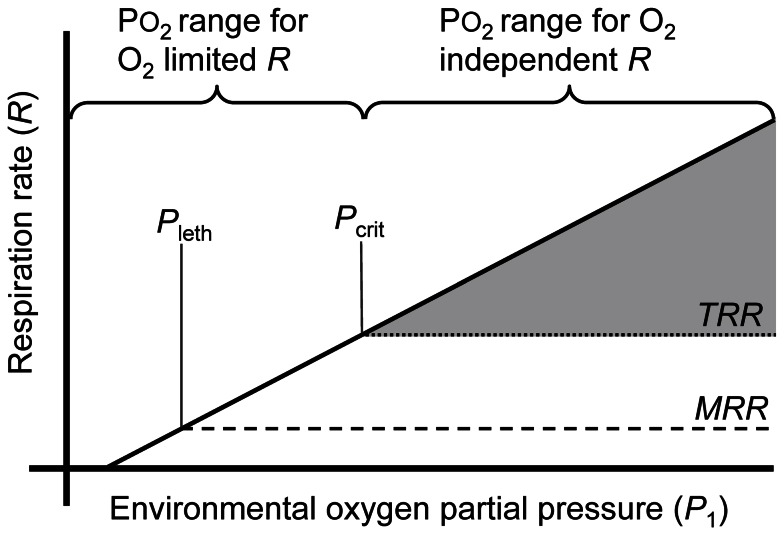
Relationship between an organism’s respiration rate and the environmental oxygen partial pressure. In this conceptual diagram, the solid line represents the linear relationship between environmental oxygen partial pressure and an organism’s theoretical maximum respiration rate, as predicted by Fick’s Law First of Diffusion (Eq. 1). Critical oxygen partial pressure (*P*_crit_) is the environmental oxygen level below which an organism’s theoretical maximum respiration rate is lower than its normal, or target respiration rate (*TRR*, dotted line). Respiration rate is oxygen limited when environmental oxygen partial pressure is below this critical partial pressure. Above the critical partial pressure respiration rate is generally independent of environmental oxygen (indicated by gray shading). Lethal oxygen partial pressure (*P*_leth_) is the environmental oxygen level below which an organism’s theoretical maximum respiration rate is lower than its minimum survivable (non-lethal) respiration rate (*MRR*, dashed line). Hypoxia-induced mortality will increase when environmental oxygen partial pressure is below this lethal oxygen partial pressure.

Given the prevalence of coastal hypoxia worldwide, it is useful to consider the biological and physiological basis for how low oxygen affects aquatic organisms. This is an essential step toward predicting individual, population, community, and ecosystem level effects of hypoxia. Below, we review how environmental oxygen level can be linked to an organism’s respiration rate, and define oxygen thresholds below which sub-lethal and lethal effects of hypoxia can be expected occur. In a low oxygen environment, the rate at which oxygen is delivered to an organism may limit its respiration rate and metabolic activity. This rate of oxygen delivery can be expressed using Fick’s First Law of Diffusion, which describes the flow of oxygen or other gas diffusing across a respiratory membrane (e.g., integument, gills, or lungs) as:
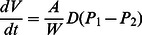
(1)where *V* is volume of oxygen, *t* is time, *A* is membrane area, *W* is membrane thickness, *P*_1_ and *P*_2_ are the oxygen partial pressures on either side of the membrane, and *D* is the diffusion coefficient. For a constant diffusion coefficient (*D*), Eq. 1 predicts that as environmental oxygen partial pressure (*P*_1_) decreases, an organism could adjust to maintain internal oxygen delivery by re-shaping the respiratory membrane (i.e., increasing *A* or decreasing *W*), or by decreasing internal oxygen partial pressure (*P*_2_) (e.g., through increased heart rate or internal oxygen binding capacity). However, at the physical limits of such adjustments, the maximum potential rate of internal oxygen delivery, and thus respiration, will be linearly related to external (environmental) oxygen partial pressure (Fig. 1, solid line). The environmental oxygen level below which an organism can no longer obtain sufficient oxygen to support a normal, or target respiration rate (*TRR*; Fig. 1, dotted line) is often termed the organism’s critical oxygen partial pressure (*P*_crit_). Respiration rate (*R*) will be independent of environmental oxygen above *P*_crit_, and will be limited by and proportional to environmental oxygen below *P*_crit_ (Fig. 1). Expanding on this concept, the environmental oxygen level below which an organism can no longer obtain sufficient oxygen to support a minimum survivable (non-lethal) respiration rate (*MRR*; Fig. 1 dashed line) can be thought of as the organism’s lethal oxygen partial pressure *P*_leth_. Below *P*_leth_, there will be an increased probability of mortality due to low environmental oxygen.

The calanoid copepod *Acartia tonsa* (Dana) has been subject of a number of studies describing its responses to hypoxia. Under experimental hypoxic conditions *A. tonsa* showed decreased egg production, egg hatching success, and somatic growth [16], [17], [18], and severe hypoxia (<1.2 mg L^−1^, ∼0.9 mL L^−1^ or ∼3.3 kPa) resulted in mortality [14], [15]. These and other studies provide a strong foundation for a synthesis describing how *A. tonsa* is affected by low environmental oxygen. We hypothesized that *A. tonsa* egg production, somatic growth, and ingestion rates would all respond in a similar manner to low environmental oxygen, with all three being related to oxygen dependent changes in respiration rate. Based on available published and newly acquired experimental data, the relationship between respiration rate and environmental oxygen proposed in Fig. 1 was parameterized and evaluated for *A. tonsa*. Based on this relationship, we derived a model predicting the effects of hypoxia on *A. tonsa* egg production, somatic growth, and mortality. This predictive model can be used to better understand individual and population levels effects of hypoxia on this widespread coastal zooplankton species, and the approach described here to define critical and lethal oxygen levels can also be applied to characterize the effects of hypoxia on other aquatic organisms.

## Methods

### Supplementary Ingestion Experiments

To supplement the available published data describing the effects of low oxygen on *A. tonsa* egg production and somatic growth, we conducted a series of feeding experiments in which ingestion rates were measured under normoxic and hypoxic conditions. In three separate experiments, 12 bottles were incubated, three normoxic and three hypoxic treatments with copepods, and three normoxic and three hypoxic copepod free controls. *A. tonsa* used in these experiments were collected from the Chesapeake Bay and reared in the laboratory for several generations. Collection activities did not involve endangered or protected species and were not conducted in privately-owned or protected locations, and no specific permits were required for the described study. Water from the Choptank River (salinity 10) was GF/F filtered for use in experiments. Dissolved oxygen was measured with a calibrated YSI model 85 dissolved oxygen meter, measuring oxygen in units of % saturation, which can be expressed in kPa following a simple linear conversion (http://water.usgs.gov/software/DOTABLES/). Hypoxic water was created by purging Choptank River water with N_2_ gas until the desired low oxygen partial pressure was reached (2.4 kPa = 1 mg L^−1^ = 0.7 mL L^−1^), a method used in other similar experiments [15], [16]. Hypoxic water was then carefully transferred to 610 mL clear polycarbonate bottles, dissolved oxygen was measured again to verify initial oxygen conditions (range from 2.2 to 3.8 kPa across all hypoxic bottles and all experiments, all normoxic bottles were near atmospheric equilibrium), and *Rhodomonas* sp. culture in exponential growth phase was added to each bottle (mean initial concentration 1921 cells mL^−1^, +/−890 SD). Based on an estimated *Rhodomonas* sp. carbon content of 55 pg C cell^−1^ [19], this resulted in a mean concentration of 106 *µ*g C L^−1^, within the range of *ca.* 50–400 *µ*g C L^−1^ where *A. tonsa* exhibits maximum clearance rates [20], [21]. Ten to fifteen adult female *A. tonsa* were added to each treatment, and bottles were sealed with paraffin film to exclude atmospheric gas. Bottles were incubated on a rotating plankton wheel (three revolutions per minute) for 1–2 d (2 d in first experiment; 1 d in others) at 18.5°C under a 12 h:12 h light:dark cycle and low light (20 *µ*mol photons m^−2^ s^−1^). At the end of incubations, final dissolved oxygen was measured in each bottle (range from 7.2 to 8.2 kPa across all hypoxic bottles and all experiments, all normoxic bottles were near atmospheric equilibrium) and copepods were enumerated and vital status checked. Initial and final algal concentrations were measured on unpreserved samples using a Coulter particle counter, with count validation done on a subset of samples using a settling chamber with light microscope. Copepod ingestion rates (cells copepod^−1^ d^−1^) were calculated [22], and converted to dry wt specific carbon ingestion (*µ*g C mg dry wt^−1^ d^−1^) using a *Rhodomonas* sp. carbon content of 55 pg C cell^−1^ [19] and adult female *A. tonsa* dry wt of 4.045 *µ*g at 18.5°C [23]. The *Rhodomonas* food culture was grown at the same temperature and light regime as experiments, but with irradiance of approximately 60 *µ*mol photons m^−2^ s^−1^, and with GF/F filtered water amended with F/2 media.

### Relating Observed Rates to Environmental Oxygen

A primary goal of this study was to evaluate the responses of *A.* *tonsa* egg production, somatic growth, and ingestion rates to environmental oxygen, and to interpret the results with reference to the effects of low oxygen on respiration rate (Fig. 1). To accomplish this, we compiled experimental data for *A. tonsa* under low environmental oxygen, including measurements of egg production rate (eggs female^−1^ d^−1^; [16], [18], [24]), somatic growth rate (mm^3^ copepod^−1^ d^−1^; [16]), and ingestion rate (*µ*g C mg dry wt^−1^ d^−1^; this study). To allow direct comparisons among these different rates, and to relate them to respiration rate, all rate data were converted to the analogous units in terms of oxygen utilization (*µ*g O_2_ mg dry wt^−1^ d^−1^), using literature reported conversion factors specific to *A. tonsa* (Table 1). Details of how each type of measurement was converted to units of oxygen utilization are given in Appendix S1. The result, after conversion, was a dataset of egg production, somatic growth, and ingestion rate measurements expressed in units of the corresponding rate of oxygen utilization (*µ*g O_2_ mg dry wt^−1^ d^−1^), which we termed the analogous respiration rate (*ARR*). Some of the data used were reported in the literature only as means at a specific oxygen partial pressure or within a specific experiment. Therefore, all *ARR* values were averaged by the environmental oxygen partial pressure at which they were measured and by study, in order to maintain consistency and comparability across the dataset.

**Table 1:** Factors used in the conversion of *Acartia tonsa* egg production, somatic growth, and ingestion rates to analogous respiration rates (*ARR*, *µ*g O_2_ mg dry wt^−1^ d^−1^).

Description	Value	Units	Reference
egg dry wt	0.104	*µ*g dry wt egg^−1^	[20]
adult female dry wt	dry wt* = *8.67–0.25 *T*	*µ*g dry wt female^−1^	[23]
respiratory cost of egg production	264	*µ*g O_2_ mg egg dry wt^−1^	[20]
volume to dry wt conversion	167.6	*µg* dry wt mm^−3^	[25]
dry wt to carbon weight conversion	0.4	*µ*g C *µ*g dry wt^−1^	[26]
average dry wt: nauplius to CIII	Temp: 25°C, O_2_: high = 0.336, intermediate = 0.354, low = 0.283;Temp: 15°C, O_2_: high = 0.393,intermediate = 0.416, low = 0.334	*µ*g dry wt copepod^−1^	[16]
net growth efficiency, and relationship torespiration and growth rates	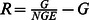 *NGE* = 0.75,	*µ*g C mg dry wt^−1^ d^−1^	[20]
respiratory quotient (0.9) expressed in units of mass	0.338	*µ*g C *µ*g O_2_^−1^	[27]
relationship between ingestion and respiration	*R* = 0.07 *I* +33.39	*µ*g C mg dry wt^−1 ^d^−1^	[20]
salinity dependent Q_10_; linearly interpolated forsalinity between reported values; closest value usedfor outside reported range	salinity 15 = 1.5; salinity 25 = 2.03;salinity 35 = 2.22	None	[28]

*T* is experimental temperature (°C ), *R* is respiration rate (*µ*g O_2_ mg dry wt^−1^), *NGE* is net growth efficiency, *G* is somatic growth rate, *I* is ingestion rate.

The responses of *A. tonsa* egg production, somatic growth, and ingestion to environmental oxygen were then evaluated by applying the concepts illustrated in Fig. 1, plotting compiled analogous respiration rates against their associated experimental oxygen partial pressures, and testing for a linear relationship between the two under low environmental oxygen conditions. To determine the oxygen thresholds for sub-lethal and lethal effects of hypoxia (Fig. 1, *P*_crit_ and *P*_leth_, respectively), we used published measurements of *A. tonsa* respiration rates in environments with ample oxygen, including those for animals under natural conditions (*reviewed in* [29]) and those for persistently starved animals [20], [30]. Respiration under natural conditions was used to estimate *A. tonsa* target respiration rate (Fig. 1, *TRR*), and respiration under persistent starvation was used to estimate minimum survivable respiration rate (Fig. 1, *MRR*). To parameterize the increase in hypoxia-induced mortality below the lethal oxygen threshold (*P*_leth_), we used published 24-h survival experiments for *A. tonsa* at various levels of low oxygen [15].

## Results

### Supplementary Ingestion Experiments

**Figure 2:**
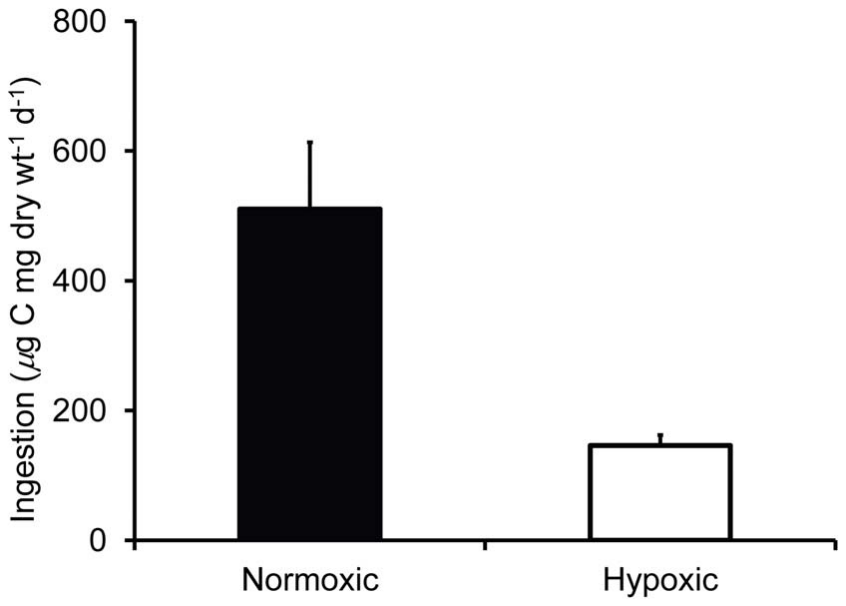
Feeding by *Acartia tonsa* in normal and low oxygen water. Ingestion rates of adult female *Acartia tonsa* under incubation conditions of hypoxia (mean oxygen 5.0 kPa = 2.1 mg L^−1^ = 1.6 mL L^−1^) and normoxia (mean oxygen 19.9 kPa = 8.3 mg L^−1^ = 6.2 mL L^−1^). Data are graphed as means of three separate laboratory feeding experiments where animals were fed *Rhodomonas* sp. and incubated for 1–2 d. Error bars are+SD.

The average oxygen partial pressure during ingestion experiments was 5.0 kPa, ±1.9 SD (2.1 mg L^−1^ = 1.6 mL L^−1^) in the hypoxic bottles and 19.9 kPa, ±1.3 SD (8.3 mg L^−1^ = 6.2 mL L^−1^) in the normoxic bottles, based on initial and final oxygen measurements. Algal growth rates were not significantly different in normoxic and hypoxic control bottles (2-sample *t*-test, *t* = −0.18, *p* = 0.86, df* = *14). Therefore, ingestion rates were calculated using average algal concentrations from all controls in each experiment. Mean ingestion rate in the normoxic bottles was 511 *µ*g C mg dry wt^−1^ d^−1^, and this was significantly higher than the 146 *µ*g C mg dry wt^−1^ d^−1^ ingestion rate in the hypoxic bottles (Fig. 2; 2-sample *t*-test, *t* = −6.06, *p = *0.026, df* = *2).

### Relating Observed Rates to Environmental Oxygen

**Figure 3:**
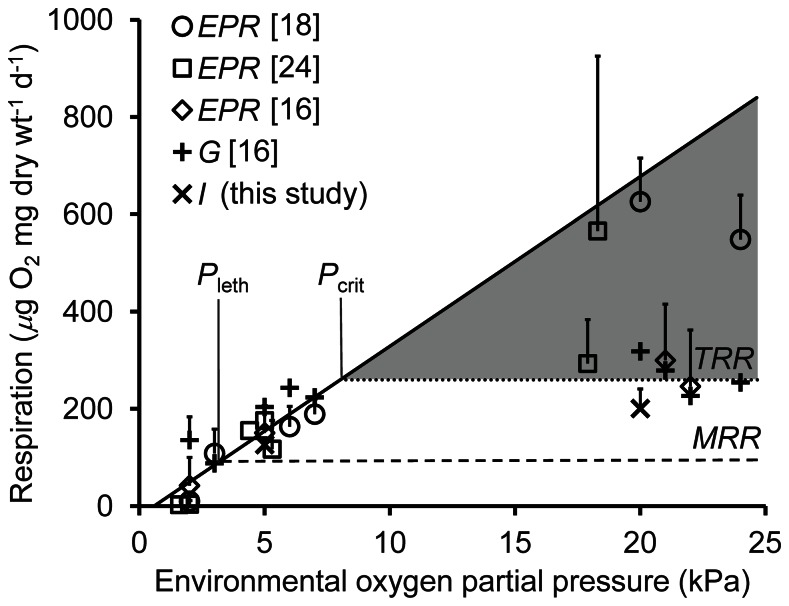
Relationship between *Acartia tonsa* respiration rate and environmental oxygen partial pressure. Using the same concepts illustrated in Fig. 1, analogous respiration rates (*ARR*) are plotted against associated environmental oxygen partial pressures. Theoretical maximum respiration rate (solid black line) is the linear regression through all data <8 kPa oxygen partial pressure. Target respiration rate (*TRR*, dotted line) is the average of normal respiration rates for *A. tonsa* (from 10 studies reviewed in [29]). Minimum survivable respiration rate (*MRR*, dashed line) is the average of respiration rates of persistently starved *A. tonsa* (reported in [20], [30]). *A. tonsa* critical oxygen partial pressure (*P*_crit_, based on *TRR*) and lethal oxygen partial pressure (*P*_leth_, based on *MRR*) are also indicated. Gray shading indicates the region of oxygen independent respiration. Each type of symbol indicates a specific data source (citations on graph) and whether the measurement was of egg production rate (*EPR*), somatic growth rate (*G*), or ingestion rate (*I*). Error bars are+SD. *ARR* values are means at each distinct oxygen partial pressure and within each study, and the linear regression is based on 17 mean *ARR* values from a total of 153 individual measurements (*see text for details*). Regression statistics: y = 34.9×–20.5; R^2^ = 0.735; slope *p*-value <0.0005; *n = *17.



At normal environmental oxygen partial pressures (>15 kPa), analogous respiration rates (*ARR*) from *A. tonsa* egg production, somatic growth, and ingestion were higher and more variable than at lower environmental oxygen (Fig. 3). At low oxygen (<8 kPa), *ARR* values were lower and fell within a much more narrow range, consistent with the conceptual relationship proposed in Fig. 1. Also, under low oxygen *ARR* showed a statistically significant linear increase with increasing oxygen (R^2^ = 0.735, *t = *6.41, *p*<0.0005), as indicated by least squares regression through all data points <8 kPa oxygen (Fig. 3, solid black line). This regression was based on 17 mean *ARR* values, which in turn were calculated from a total of 153 individual measurements of egg production, somatic growth, and ingestion. The resulting regression line is an estimate of the theoretical maximum respiration rate of *A. tonsa* as a function of oxygen partial pressure (corresponding to the solid line in Fig. 1), and is described by the equation:

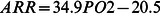
(2)where *ARR* is analogous respiration rate (*µ*g O_2_ mg dry wt^−1^ d^−1^) and PO_2_ is environmental oxygen partial pressure (kPa).

To calculate the target respiration rate for *A. tonsa* (Fig. 3, *TRR*), we averaged the non-oxygen limited respiration rates of animals under natural conditions from 10 separate studies (*reviewed in* [29]). The resulting *TRR* (261.5 *µ*g O_2_ mg dry wt^−1^ d^−1^) corresponds to a critical oxygen partial pressure (*P*_crit_) of 8.1 kPa (±1.7 kPa SE), approximately 3.1 mg L^−1^ = 2.3 mL L^−1^, which is specific to 18°C since all data were standardized to this temperature. Using reported Q_10_ values [28], temperature specific *P*_crit_ can be calculated as:
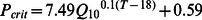
(3)


where Q_10_ is assigned as described in Table 1 and *T* is temperature (°C).

To calculate the minimum survivable respiration rate for *A. tonsa* (Fig. 3, *MRR*), we averaged the respiration rates of starved animals reported in two studies [20], [30]. *A. tonsa* respiration rate decreases under chronic starvation [20], [30], and we make the assumption that this lower starved respiration rate approximates the minimum respiration rate at which the animals can still survive (*MRR*). Averaging the mean starved respiration rates from these two study [20], [30], the resulting *MRR* (91.0 *µ*g O_2_ mg dry wt^−1^ d^−1^) corresponds to a lethal oxygen partial pressure (*P*_leth_) of 3.2 kPa (±1.7 kPa SE), or approximately 1.2 mg L^−1^ = 0.9 mL L^−1^. This also is specific to 18°C, and temperature specific *P*_leth_ can be calculated as:
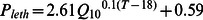
(4)where Q_10_ is assigned as described in Table 1 and *T* is temperature (°C). To parameterize the increase in hypoxia-induced mortality below *P*_leth_, we used a published experimental study of *A. tonsa* survival over 24 h at low oxygen. The referenced study [15] found that *A. tonsa* mortality increased incrementally below 3.7 kPa, and reached 100% mortality at half this oxygen (1.85 kPa) (for comparison, Eq. 4 yields *P*_leth_ = 3.6 kPa at study temperature and salinity conditions). Accordingly, we considered the probability of hypoxia-induced mortality to be 0% at *P*_leth_, and to increase to 100% at one half of *P*_leth_. At 18°C, this corresponds to no mortality at 3.2 kPa (*P*_leth_) and 100% mortality at 1.6 kPa. Expressed in terms of respiration, this is 0% mortality for *MRR* (91.0 *µ*g O_2_ mg dry wt^−1^ d^−1^) and 100% mortality for *ARR* at 1.6 kPa (35 *µ*g O_2_ mg dry wt^−1^ d^−1^, Eq. 2). Linearly interpolating between these *ARR* endpoints, the temperature dependent probability of hypoxia-induced mortality within 24 h (*m*) can be calculated as:
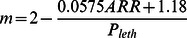
(5)where ARR is from Eq. 2, Pleth is from Eq. 4, and m≥1.0 is equivalent to 100% mortality. Resulting estimates of m should be considered specific to copepodites, since survival of A. tonsa nauplii has been observed to remain high at lower PO2 than for copepodites [15].

Using Eqs. 2–5 and the conversion factors in Table 1, we derived a model for predicting the sub-lethal and lethal effects of hypoxia on *A. tonsa* based on environmental temperature and oxygen partial pressure (Table 2). Critical oxygen partial pressure (*P*_crit_) is first calculated from the temperature (*T*) of the water in which a copepod resides, with Q_10_ also dependent on salinity (Table 1). When environmental oxygen partial pressure is less than *P*_crit_, Eq. 2 can be used to calculate the copepod’s analogous respiration rate (*ARR*). Subsequently, *ARR* can be used to calculate rates of somatic growth and egg production. Lethal oxygen partial pressure (*P*_leth_) is also calculated from temperature and salinity-specific Q_10_; when environmental oxygen partial pressure is less than *P*_leth_ then *ARR* can be used to calculate the probability of hypoxia-induced mortality (*m*). It is important to note that the equations for egg production, somatic growth, and mortality rates in Table 2 should not be used when environmental oxygen partial pressure is greater than the calculated critical and/or lethal partial pressures, since these relationships are only applicable when oxygen limits respiration rate.

**Table 2:** Summary of the model predicting effects of hypoxia on *Acartia tonsa* vital rates.

Variable	Formula	Units	Source
Oxygen partial pressure (PO_2_)	none	kPa	measured or otherwise determined
Temperature (*T*)	none	°C	measured or otherwise determined
Q_10_	none	none	Table 1, [28]
Critical partial pressure (*P*_crit_)	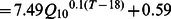	kPa	Eq. 3
Analogous respiration rate (*ARR*)	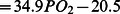	*µ*g O_2_ mg dry wt^−1^ d^−1^	Eq. 2
Egg production rate	= 0.00379 *ARR*	*µ*g dry wt *µ*g dry wt^−1^ d^−1^	Table 1, [20]
Somatic growth rate	= 1.014 *ARR*	*µ*g C mg dry wt^−1^ d^−1^	Table 1, [20]
Lethal partial pressure (*P*_leth_)	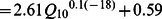	kPa	Eq. 4
24-h probability of mortality (*m*) for copepodites	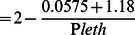	d^−1^	Eq. 5

Critical oxygen partial pressure (*P*_crit_) is calculated from temperature. When environmental oxygen partial pressure (PO_2_) is less than *P*_crit_ then Eq. 2 can be used to calculate analogous respiration rate (*ARR*), which can then be used to calculate rates of egg production and somatic growth. Lethal oxygen partial pressure (*P*_leth_) is also calculated from temperature. When PO_2_ is less than *P*_leth_ then *ARR* can be used to calculate the probability of hypoxia-induced mortality (*m*), where *m* ≥1.0 is equivalent to 100% mortality.

**Figure 4:**
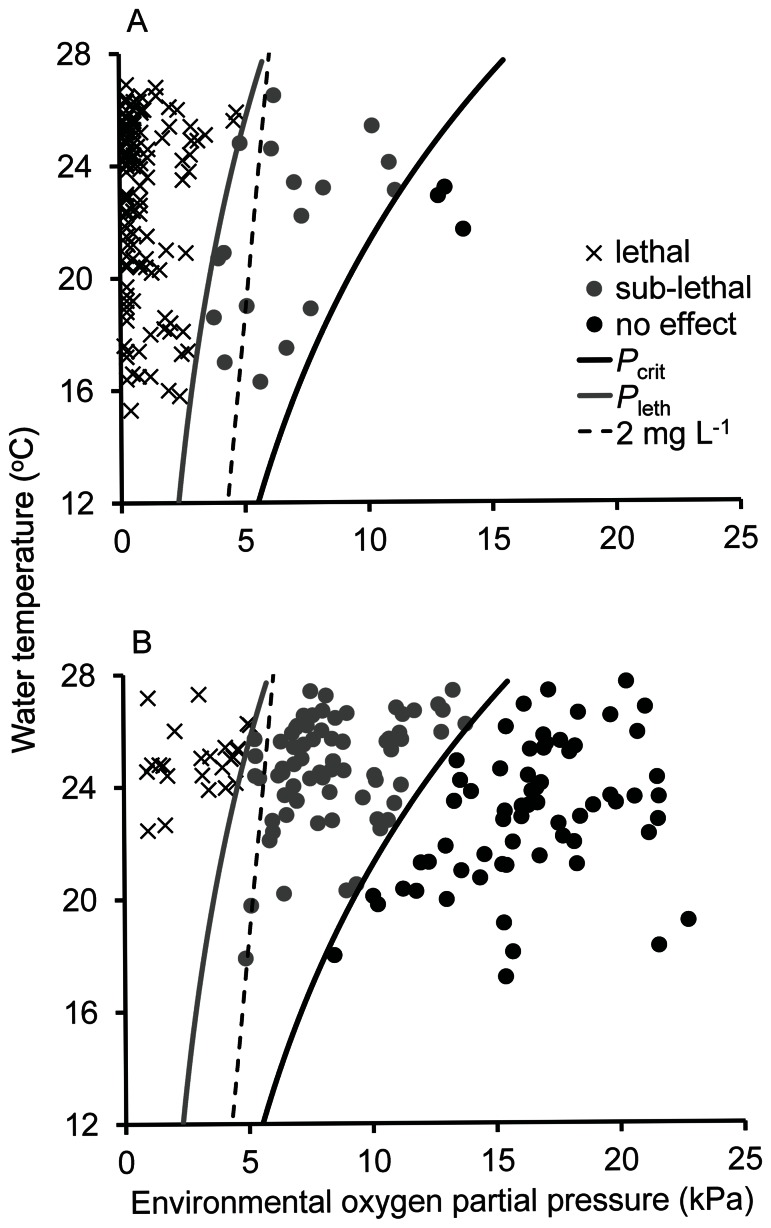
Model predicting the influence of hypoxia on *Acartia tonsa* applied to data from Chesapeake Bay. Each data point is a measurement of temperature and oxygen partial pressure between June and September in the sub-pycnocline Chesapeake Bay. Points are coded to indicate predicted lethal, sub-lethal, or no effect on *Acartia tonsa*. The temperature and oxygen measurements used were taken monthly from 1986 to 2011 as part of Chesapeake Bay Program (CBP) monitoring (http://www.chesapeakebay.net/data). Thresholds for critical (*P*_crit_) and lethal (*P*_crit_) oxygen partial pressures are also indicated, as is the traditionally hypoxic 2 mg L^−1^ oxygen facet. Data are from (a) a region with severe summer hypoxia (CBP station CB4.3C), and (b) a region outside of the extent of severe hypoxia (CBP station CB6.1C).

To illustrate a practical application of the model described in Table 2, we predicted *P*_crit_ and *P*_leth_ in relation to measurements of oxygen and temperature in the sub-pycnocline regions of the Chesapeake Bay during summer (Fig. 4), comparing a northern station that typically experiences severe summer hypoxia to a southern station where hypoxia is much less severe. *A. tonsa* residing below the pycnocline at the northern station were predicted to have experienced negative effects of hypoxia in 98% of samples, and lethal effects in 89% of samples (Fig. 4a). Conditions at the southern station suggested a much less detrimental sub-pycnocline environment. However, negative effects were still predicted in 60% of samples, and lethal effects in 16% of samples (Fig. 4b). Fig. 4 also shows the approximate location of a commonly used definition of hypoxia (<2 mg L^−1^, dashed line). Although all samples with predicted lethal effects fell below this standard of hypoxia, many samples with oxygen concentrations higher than 2 mg L^−1^ were predicted to have sub-lethal effects on *A. tonsa*.

## Discussion

Reduced feeding is a commonly reported effect of hypoxia on aquatic animals including zooplankton [2], [4], [31], and our results suggest that it occurs for *A. tonsa* (Fig. 2). Under hypoxic conditions (oxygen <*P*_crit_), oxygen rather than food intake determines respiration rate, and high ingestion rates could lead to intake in excess of food requirements. *A. tonsa* stores little in the way of reserve energy [32], and is unlikely to benefit from feeding in excess of short-term energetic demands. Such superfluous feeding could even be disadvantageous, since feeding activity can increase risk of predation in zooplankton [33]. Feeding also requires energy, and although the energetic costs of *A. tonsa* feeding are small relative to those of growth and assimilation [20], these costs might still be non-trivial during periods when low oxygen limits respiration rate, at which times elevated consumption cannot result in more useable energy from respiration. Therefore, reduced feeding under low oxygen likely represents an ecologically advantageous behavior.

The conceptual relationship between respiration rate and environmental oxygen partial pressure that is illustrated in Fig. 1 is similar to that reported for various other aquatic animals [8], [34], [35]. To evaluate the applicability of this relationship to *A. tonsa*, we used analogous respiration rates (*ARR*) derived from *A. tonsa* egg production, somatic growth, and ingestion rates measured in four separate studies ([16], [18], [24] and the present study), as well as direct *A. tonsa* respiration rate measurements from 12 studies ([20], [30] and 10 studies *reviewed in* [29]). Our results suggest that this relationship is useful for predicting *A. tonsa* respiration rate at low oxygen, and therefore, ultimately for predicting the effects of hypoxia on *A. tonsa* egg production, somatic growth, and mortality. As would be expected from Fig. 1, *ARR* varied widely and appeared to be independent of oxygen at high environmental oxygen (>15 kPa). However, values also typically fell within the expected range (Figs. 1, 3, gray shaded areas), being less than or equal to the theoretical maximum respiration rate (Fig. 3, solid black line), but near or above the average target respiration rate (Fig. 3, dotted line). Also as expected from Fig. 1, *ARR* was much less variable and appeared to be linearly dependent on oxygen at low environmental oxygen (<8 kPa), where a linear relationship explained >73% of the variation in average *ARR* values (Fig. 3, R^2^ = 0.735). A comparable degree of certainty can also be extended to model predictions for the effects of hypoxia on egg production and somatic growth (Table 2), since these *ARR* values came directly from measurements egg production and somatic growth rates, and the same factors used to convert these original measurements to *ARR* (Table 1) are then used to back-convert *ARR* to egg production and somatic growth rates (Table 2).

Our estimates of lethal (*P*_leth_) and critical (*P*_crit_) oxygen partial pressures appear to agree well with the results from other studies. In low oxygen survival experiments conducted at 20°C and 30 salinity, *A. tonsa* survival decreased significantly between 3.7 kPa and 3.3 kPa oxygen partial pressure [15]; in our study 3.6 kPa was the lethal oxygen partial pressure that Eq. 4 yields for the same temperature and salinity conditions. Previous work [7] reviewed *P*_crit_ estimates for oceanic species, finding values ranging from <0.2 kPa to around 6 kPa, with measurements made mostly at 5°C. At 5°C, and assuming a Q_10_ of 2.03 (Table 1), Eq. 3 suggests a *P*_crit_ of 3.6 kPa for *A. tonsa*, well within the range of reported *P*_crit_ values [7], [8]. This *P*_crit_ is also near to the 4 kPa value proposed to distinguish animals inhabiting mainly high oxygen environments (higher *P*_crit_ values) from those displaying specific adaptations for inhabiting low oxygen environments (lower *P*_crit_) [8]. Behavioral adaptations for avoiding hypoxia have been demonstrated in Chesapeake Bay *A. tonsa* populations, suggesting that, given the short generation time of *A. tonsa*, evolution of hypoxia specific adaptations could be possible within the span of approximately 50–250 years [10]. Thus, it is possible that some *A. tonsa* populations have hypoxia specific adaptations, but no evidence of physiological adaptations has yet been reported [9].

The *P_crit_* estimates made in this study were based on a target respiration rate (*TRR*) that was the average of several direct measurements of *A. tonsa* respiration rate (*reviewed in* [29]). These measurements were taken on healthy copepods maintained under pseudo-natural conditions, so that resulting measurements should be comparable to natural respiration rates. Therefore, *P_crit_* estimated in our study (Fig. 3, Eq. 3) should be generally applicable to *A. tonsa* in nature. However, in future application of the proposed predictive model (Table 2), an alternative to using Eq. 3 would be to measure *P_crit_* value(s) specifically for the study area. Measured *P_crit_* could then be substituted for Eq. 3 predictions and the model otherwise used exactly as described.

Our study also highlights the potential importance of temperature in regulating the effects of hypoxia on the copepod *Acartia tonsa*, and on aquatic organisms in general. As environmental temperature increases a lesser degree of oxygen depletion is required to have deleterious effects on *A. tonsa* (Fig. 4). The same type of response applies to all ectotherms, for which respiration rate increases with environmental temperature and body temperature. *A. tonsa* has been reported to occur at temperatures from −1°C to 32°C [36]. Assuming a Q_10_ of 2.03, this temperature range corresponds to a *P*_crit_ between 2.5 and 20.8 kPa (∼1.5 to 6.2 mg L^−1^ = 1.1 to 4.8 mL L^−1^). Thus, at the upper end of its temperature distribution (32°C) *A. tonsa* could experience oxygen limited respiration at slightly below atmospheric oxygen saturation (21.2 kPa). This suggests a possible role of oxygen limitation in determining this copepod’s ecological upper temperature threshold, as appears to apply to many marine ectotherms (*reviewed in* [37]).

In this study, we developed a model predicting the sub-lethal and lethal effects of hypoxia on the copepod *Acartia tonsa.* Environmental oxygen partial pressure is used to predict respiration rate, assuming that the two are linearly related at low environmental oxygen as would be predicted by Fick’s First Law of Diffusion (Eq. 1). Respiration rate is then linked to other processes (egg production rate, somatic growth rate, and probability of mortality) based on published studies of *A. tonsa* bioenergetics and survival under low oxygen conditions. When combined with information on copepod vertical migration into and out of hypoxic water, rate estimates from Table 2 could be used to incorporate the effects of hypoxia into population, community or ecosystem level models that include *A. tonsa*. This type of approach could also be used to describe the responses of other aquatic species to low environmental oxygen. Given the prevalence of hypoxia in coastal systems worldwide and the increased use of mathematical models in environmental sciences, such approaches will be valuable for better understanding and predicting the potential ecological impacts of hypoxia.

## Supporting Information

Appendix S1 **Detailed description of how each type of measurement (** ***Acartia tonsa*** **egg production, somatic growth, and ingestion rate) was converted to units of oxygen utilization, or analogous respiration rate (** ***ARR*** **).**
(DOCX)
