# Supplementary material for: Predicting the Effects of Coastal Hypoxia on Vital Rates of the Planktonic Copepod Acartia tonsa Dana
Source: PLoS One. 2013 May 17;8(5):e63987. doi: 10.1371/journal.pone.0063987 (PMC3656935; doi:10.1371/journal.pone.0063987)
Supplement: Appendix S1 — Detailed description of how each type of measurement ( Acartia tonsa egg production, somatic growth, and ingestion rate) was converted to units of oxygen utilization, or analogous respiration rate ( ARR ). (DOCX) [file pone.0063987.s001.docx]

To make direct quantitative comparisons among the reported sub-lethal effects of hypoxia on *A. tonsa* (reductions in egg production, somatic growth, and ingestion), it was necessary that all rates be standardized to the same units. In the proposed model (Fig. 1), the process linking these rates is respiration rate; therefore, all data were standardized by conversion to analogous respiration rates (*ARR*, *µ*g O_2_ mg dry wt^-1^ d^-1^). These *ARR* values represent the respiration rate predicted to occur at a given (measured) egg production, somatic growth, or ingestion rate. Table 1 lists the bioenergetics parameter values and conversion factors that were used to calculate *ARR*.

The rate data used to calculate *ARR* values were all from original studies that reported in a variety of units, all of which were converted to *ARR* (*µ*g O_2_ mg dry wt^-1^ d^-1^) as described in this appendix. Measured rates (egg production, somatic growth, and ingestion) were converted directly to *ARR* using published conversion factors to empirically relate each to respiration rate, and these conversion factors were derived from experimentally observed relationships between each measured rate and measured respiration rate. Therefore, additional factors such as % food assimilated and excretion were implicitly accounted for when converting to *ARR*, since these processes occurred in the experimental animals on which respiration and other rates were measured. Details of each unit conversion are as follows:

- For egg production rates, the conversion factor used was the respiratory cost of *A. tonsa* egg production. The data on egg production (eggs female^-1^ d^-1^; [16,18,24]) were converted to dry wt specific egg production (*µ*g egg dry wt *µ*g female dry wt^-1^ d^-1^) using a published value for egg dry wt and published temperature specific adult female dry wt estimates (Table 1, [20,23]). These were then converted to *ARR* using the published respiratory cost of *A. tonsa* egg production (Table 1, [20]).
- For somatic growth rates, the conversion factor used was the net growth efficiency of *A. tonsa*, as related to growth and respiration rates. The data on somatic growth were reported as increases in copepod body volume over measured time increments for development from the egg stage to the CIV stage (mm^3^ copepod^-1^ d^-1^; [16], their figs. 3, 4, and 5). These were converted to dry wt specific growth rates (*µ*g dry wt copepod^-1^ d^-1^) using the published density estimate for an *A. tonsa* copepod (volume to dry wt conversion, Table 1, [25]), and then to carbon specific growth rates (*µ*g C copepod^-1^ d^-1^) using a published dry wt to carbon weight conversion (Table 1, [26]). Rates were then converted to dry wt specific carbon growth rates (*µ*g C mg *DW*^-1^ d^-1^) using the time weighted average of copepod dry wt over the naupliar and first three copepodite stages (average dry wt: nauplius to CIII, Table 1), values for which were computed from the reported dry wt (from body volume) of the animals used in the growth experiments ([16], their fig. 5]) and by assuming isochronal development for *A. tonsa* [25]. These dry wt specific carbon growth rates were converted to carbon utilized in respiration based on published *A. tonsa* net growth efficiency (Table 1, [20]) and the relationship between respiration, growth, and net growth efficiency; then to rate of oxygen utilization (*ARR*) based on published *A. tonsa* respiratory quotient (Table 1, [27]).
- For ingestion rates, the conversion factor used was the relationship between *A. tonsa* respiration rate and ingestion rate, as related to specific dynamic action. Ingestion rates measured in the present study (*µ*g C mg *DW*^-1^ d^-1^) were converted to carbon utilized in respiration based on the published relationship between ingestion and respiration (Table 1, [20]). These values were converted to rate of oxygen utilization (*ARR*) based on published *A. tonsa* respiratory quotient (Table 1, [27]).

Using the published salinity dependent Q_10_ for *A. tonsa* (Table 1, [28]), all calculated *ARR* values were then standardized to 18 °C and 27 salinity, the experimental conditions of the study in which many of conversion factors in Table 1 were measured [20]. This standardization of *ARR* allowed direct comparison of egg production, somatic growth, and ingestion rates derived from multiple studies and including experiments at multiple temperature and salinity conditions. Finally, the factors described above for converting original rate data to *ARR* (Table 1) were then used in Table 2 to directly derive the equations for converting *ARR* back to egg production and somatic growth rates, and Eq. 5 applied to predict probability of mortality from *ARR*.
